# Key Factors behind the Dynamic Stability of Pairs of Egyptian Vultures in Continental Spain

**DOI:** 10.3390/ani13172775

**Published:** 2023-08-31

**Authors:** Catuxa Cerecedo-Iglesias, Joan Lluís Pretus, Antonio Hernández-Matías, Ainara Cortés-Avizanda, Joan Real

**Affiliations:** 1 Equip de Biologia de la Conservació, Departament de Biologia Evolutiva, Ecologia i Ciències Ambientals, Facultat de Biologia, Institut de la Recerca de la Biodiversitat i (IRBIO), Universitat de Barcelona, Diagonal 643, 08028 Barcelona, Spain; jpretus@ub.edu (J.L.P.); ahernandezmatias@ub.edu (A.H.-M.); jreal@ub.edu (J.R.); 2 Department of Plant Biology and Ecology, Faculty of Biology, University of Seville, Avenida Reina Mercedes 6, 41012 Seville, Spain; acortes1@us.es; 3 Estacion Biologica Doñana, CSIC, Avenida Americo Vespucio 26, Isla de la Cartuja, 41012 Seville, Spain

**Keywords:** abundance distribution, Egyptian vultures, LISA, ROAPs, spatial autocorrelation, trophic resources, supplementary feeding stations, vulture conservation, wind farms

## Abstract

**Simple Summary:**

Understanding and modelling species distribution is crucial for conservation efforts, especially in light of the global biodiversity crisis. Here, we focus on the worldwide endangered Egyptian vulture (*Neophron percnopterus*), a large long-lived raptor, in order to explore ways of developing effective conservation strategies. We analyzed interesting differences in trends at the local level within the context of a regionally stable pattern over the past two decades in Spain, one of the most important breeding areas for this vulture. Through our analysis, we discovered that the regional stability in Egyptian vulture breeding pairs was positively associated with the abundance of griffon vultures (*Gyps fulvus*) and cattle. We also found that the presence of wind farms had a negative impact on the number of breeding pairs at the local level and that factors relating to food resources had a positive effect at both local and larger scales. To effectively conserve the Egyptian vulture, management plans should adopt a hierarchical approach and address the factors influencing breeding populations at various spatiotemporal scales.

**Abstract:**

Conservation science aims to identify the factors influencing the distribution of threatened species, thereby permitting the implementation of effective management strategies. This is key for long-lived species that require long-term monitoring such as the worldwide endangered Egyptian vulture (*Neophron percnopterus*). We studied temporal and spatial variations in the distribution of breeding pairs and examined the intrinsic and anthropic factors that may be influencing the abundance of breeding territories in continental Spain. Based on the census data of breeding pairs from 2000, 2008, and 2018, we used Rank Occupancy–Abundance Profiles to assess the temporal stability of the population and identified the spatial heterogeneity through a Local Index of Spatial Autocorrelation analysis. The GLMs showed that the abundance distribution was mainly influenced by the abundance of griffon vultures (*Gyps fulvus*) and cattle at a regional scale. Nonparametric comparisons showed that the presence of wind farms had a significant negative effect on local breeding pairs abundance, but that supplementary feeding stations and food resource-related variables had a positive impact. In light of these findings, we recommend a hierarchical approach in future conservation programs involving actions promoting regional-scale food resource availability and highlight the need to address the negative impact of wind farms at local levels.

## 1. Introduction

The species–environment relationships determining the current distribution of endangered species within their geographic range are a key ecological process; therefore, examining and understanding these species–environment relationships may be essential for the development of effective conservation strategies devoted to recovering endangered species [1,2,3]. However, the study of the distribution patterns of large long-lived species presents exceptional challenges, as it requires the integration of spatial and temporal shifts in abundances [4,5]. Furthermore, species–environment relationships depend greatly on the scale at which they are studied [6,7,8], and the neglect of appropriate spatial and temporal considerations can lead to inaccuracies in forecasts of species distribution [9]. Species distribution is a dynamic phenomenon, characterized by spatial contractions and expansions over time, that is influenced by the interplay of biological, ecological, and biogeographic factors. In this context, the application of species distribution models (SDMs; see review [10]) has been widely used to study species occupancy and abundance patterns.

SDMs empirically examine species occupancy or abundance using grid-cells and the species–environment relationship in terms of intrinsic and extrinsic factors. Despite the advantages of using such methods (e.g., identifying important areas for species conservation; [11]), the consideration of variability in the temporal dimension is rarely addressed. The incorporation of the continuous temporal dimension (i.e., neither a static nor specific time interval; [10,12]) is a novel approach that allows us to use distinct ecological processes and time-dependent factors governing fluctuations in occupancy and abundance [13,14,15]. In addition, since species distribution patterns are also sensitive to factors operating in the local environment such as microclimate or food availability [16] that differ greatly from those at work at larger scales [6], SDMs require a specific spatial scale or scales under scrutiny. Additionally, landscape heterogeneity in terms of the availability of suitable breeding sites may also influence occupancy and abundance patterns [17], thereby promoting spatial aggregation and uneven distribution across a landscape [18,19].

Studying the occupancy and abundance distribution of large long-lived vertebrate species presents numerous challenges due to their wide range of different behaviors that require large interconnected habitats [20,21,22]. In this context, vultures are no exception, and their spatial and temporal distribution is often influenced by multiple, often environmental [23] and human-related [24,25] factors whose impact varies depending on scale. As long-lived birds, they exhibit late maturity and low reproductive rates, which leads to slow natural changes in population numbers over time [26].

Here, we use a novel approach to analyze the factors that influence temporal and spatial variation in the abundance distribution of breeding pairs of the long-lived Egyptian vulture (*Neophron percnopterus*), a species threatened worldwide at different local (i.e., specific 100 km^2^ areas within a landscape) and regional (i.e., larger geographic regions such as countries) spatial scales. Despite the crucial role that Egyptian vultures play in ecosystem health, they face threats such as habitat loss, persecution, electrocution, and poisoning [27]. In Spain, human activities have resulted in local extinctions [28] but, interestingly, in some regions the number of breeding territories is now increasing [29,30]. We used long-term Egyptian vulture monitoring information in one of this vulture’s main breeding areas. We aimed (1) to test whether or not Egyptian vulture occupancy and abundance has changed over time in continental Spain; (2) to determine the spatial patterns, i.e., the spatial heterogeneity, of the abundance of breeding territories in the study region; (3) to identify the factors contributing to spatial variation at the local scale; and (4) to investigate the factors responsible for changes in abundance over both time and space at the regional scale. Based on the hypothesis that both temporal and spatial factors influence species distribution, we predicted that the abundance of breeding pairs of Egyptian vultures would vary over time (i.e., a non-stationary distribution) and space (i.e., an aggregated distribution). Furthermore, we anticipated that the factors driving this species’ distribution would differ depending on the spatial scale employed [6]. The findings of this study will help develop targeted conservation plans for declining vulture populations and facilitate efforts to increase the occupancy rate of their breeding populations.

## 2. Materials and Methods

### 2.1. Study Species

**Figure 1:**
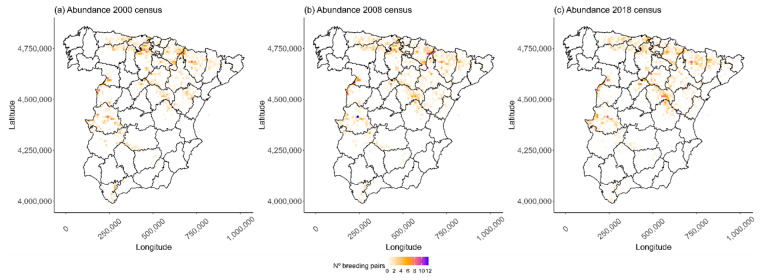
The distribution of Egyptian vulture breeding pairs in three different years: (**a**) 2000, (**b**) 2008, and (**c**) 2018. Each 100 km^2^ grid shows the number of occupied territories during the breeding period.

The Egyptian vulture is a long-lived migratory scavenger that is globally “Endangered” [27]. During the breeding period (March–August), it establishes territories in southern Europe, the Middle East, and central and southern Asia, but spends the winter in various parts of Africa. The Spanish population, which represents 12% of the world’s total [27] and 27% of the European total, suffered a serious decline in 1987–2000 [31] due to multiple causes, including poisoning [32], disturbance at breeding territories [24], electrocution [33], collision with human infrastructures such as power lines and wind turbines [34], and reduced food availability [35]. Here, we used data from the last three censuses (2000, 2008, and 2018) from continental Spain (493,719 km^2^, Figure 1), but excluded data from the Canary and Balearic Archipelagos where this vulture is a resident species [33]. Censuses were conducted using a standardized methodology in which territorial breeding pairs in potential breeding areas were searched for [36,37,38]. For each breeding territory, the location and status (occupied vs. unoccupied) was recorded. To obtain the abundance data for each census year, these locations were incorporated into a spatial Universal Transversal Mercator (UTM) grid with a resolution of 10 × 10 km and the abundance of each cell was calculated by summing the locations of confirmed breeding territories. During the analysis, we only took into account cells where the species was present in at least one year in the period 2000–2018 (n = 1033).

### 2.2. Analytical Procedure

#### 2.2.1. Analyzing the Temporal Variation in Distribution

The Rank Occupancy–Abundance Profiles (ROAPs; [39]) approach was used to test the null expectation that the regional population of Egyptian vulture can be considered stable over the years or, conversely, that significant changes have occurred (either increase or decrease) in both abundance and occupancy patterns. ROAPs are a graphical procedure based on the position of cells in a rank according to their occupancy and abundance that is similar to a classical ranking of species within communities (see [39]). They consist of scatterplots in which the X-axis (values range from 0 to 1) corresponds to the relative ranks of grid-cells based on their occupancy, and the Y-axis corresponds to the absolute abundance of pairs per grid-cell. To obtain the relative ranks, we assigned the rank position 1 to the highest abundance and divided each rank by the total number of cells (n = 1033). Three profiles were built separately from the abundance of pairs data for each 10 × 10 km cell (2000, 2008, and 2018).

Additionally, to test for differences in the occupancy and abundance distribution between the three censuses, we followed the procedure described by [39], which consists of pooling the abundance data of the three censuses and randomly assigning a year. We iterated this routine 100 times and calculated the D* statistic (the area under the curve of abundances of each year) for each run to obtain a reference random distribution. Furthermore, we compared the observed statistic D* with the random distribution and tested to see whether or not it could be considered within the scope of randomized distribution at a significance level of α = 0.05.

#### 2.2.2. Analyzing the Spatial Heterogeneity

To investigate the spatial variation in abundance, i.e., the spatial heterogeneity, we first identified the cells exhibiting aggregation patterns. To do so, we first checked for the existence of spatial autocorrelation by using the global Moran’s I test [40], a preliminary procedure for detecting at which scales a significant spatial positive dependency occurs. We further identified the cells with spatially aggregated patterns using the procedure known as the Local Index of Spatial Autocorrelation (LISA; [41]). For this two-step analysis, we analyzed the 2018, 2008, and 2000 censuses separately. Moran’s I Index reflects the degree of similarity or dissimilarity between abundance values based on the distances between the central points of the cells. The values in this index range between −1 (regular distribution, negative autocorrelation) and +1 (aggregated distribution, positive autocorrelation), zero being the reference random distribution. This index was calculated using the distance matrix between the central points of the cells that had been occupied at least once during the census (n = 1033). Then, we used a Monte Carlo simulation and 999 permutations to obtain the significance of the spatial autocorrelation at a regional level. Once we had detected the spatial autocorrelation of the abundance data, we used the LISA to detect spatial aggregation areas in which the number of breeding pairs was greater or lower than in nearby areas. The LISA measures allow us to distinguish between spatial aggregation units and non-aggregation units using the scatterplot resulting from Moran’s I Index and by dividing it into four quadrants with the abundance values plotted against spatial distances [42,43]. These values are classified according to the quadrant in which they are located on the scatterplot: High–High (high surrounded by high), Low–Low (low surrounded by low), High–Low (high surrounded by low), and Low–High (low surrounded by high). Then, we combined classified the High–High and High–Low cells as High cells, and Low–Low and Low–High cells as Low cells. High cells represent the clusters where the number of breeding pairs is significantly higher than in neighboring cells (spatial aggregation), while Low cells represent clusters in which abundances are significantly lower than the abundances in neighboring cells (spatial non-aggregation).

#### 2.2.3. Analyzing the Factors That Shape Recent Abundances at Local and Regional Scales

At local scale, once we had identified the cells with aggregated patterns, we then analyzed the factors driving this aggregation. To do so, we used a nonparametric Kruskal–Wallis test to compare each of the 16 variables relating to habitat, food availability, human pressure, and heterospecific attraction that explain the differences in abundance between the High and Low cells (Table 1). The significance level was adjusted using the Bonferroni correction.

To determine the factors that shaped the abundance distribution of Egyptian vultures in the 2018 census in continental Spain at a large scale, we performed generalized linear models (GLM; negative binomial distribution and log link function; [44]). The challenge of limited fine environmental data is a common issue in studies analyzing diverse environmental and anthropic variables across lengthy time spans. Our study encountered this limitation, with a temporal mismatch between the explanatory variable data and species abundance data collected in 2000, 2008, and 2018. Notably, data for explanatory variables were available only after 2008, such as 2009 census data for livestock and 2018 data for wind turbines and landfills. To address this, we focused our analysis on the year 2018, postulating that this later data would yield stronger models for associating Egyptian vulture distribution compared to earlier years. Additionally, we considered only explanatory variables with significant differences between High and Low cells and used the abundance of breeding pairs per cell as a dependent variable to analyze whether or not the same factors drive the abundance distribution at different scales. Moreover, in our analyses, we considered two key assumptions regarding the relationship between Egyptian vulture abundance and environmental factors. Firstly, we assumed that most of the variability in abundances observed in 2018 could be explained by the abundances registered during the previous census and therefore we considered the abundance of the previous census to be a proxy for habitat quality, based on the findings of [45]. Secondly, we incorporated a temporal term into our statistical model to account for changes in abundance distribution over time. We assumed that any independent variable (e.g., food availability) that was found to have a significant effect on abundance distribution after accounting for temporal changes was a potential driver of abundance changes between censuses. Therefore, apart from variables with differences between High and Low cells, we also considered spatial and temporal terms. The spatial term was the third-degree polynomial derived from coordinates, longitude (x), and latitude (y) of the central point of the 10 × 10 km cells in order to, on the one hand, avoid the false correlation between species and its environment and, on the other hand, to identify if there were spatial patterns in the abundance data that could not be accounted for or explained by the environmental variables [46]. The temporal terms corresponded to the abundance of breeding pairs of Egyptian vultures according to data from the 2000 (hereafter, NP00) and 2008 (hereafter, NP08) Egyptian vulture censuses. These two temporal terms were included separately in two different models.

We developed the analysis in the R environment [47] using the “adespatial” [48], “MASS” [49], and “MuMIn” packages [50]. To select the best models, we used the Corrected Akaike Selection Criterion (AICc; see the average model with a ΔAIC threshold of <2 in Appendix D; [51]).

#### 2.2.4. Explanatory Variables

Grid cells were characterized by 16 variables relating to habitat, food availability, human pressure, and heterospecific relationships to determine the factors potentially shaping the abundance distribution. Habitat was represented by land-use coverage in several different categories (see Table 1). In addition, we included elevation as a habitat-related variable since it is associated with the reproductive habitat of breeding pairs such as cliffs [26]. We used the number of cows and sheep per 10 × 10 km cell as a proxy for potential food resources following [52]. We also considered the locations of landfills and supplementary feeding stations (specific places where carcasses are deposited to feed avian scavengers to increase the availability of food resources as a vulture conservation measure; see review [53]) as predictable anthropogenic food sources [54]. Human pressure was evaluated using various sources of information, including the location of wind farms, the number of poison-related mortality events [55], and the coverage of urban areas, all of which have been shown to be relevant factors in the breeding distribution of Egyptian vultures [56]. Finally, we used the number of breeding pairs of the dominant species in the scavenger guild, the griffon vulture (*Gyps fulvus*), as a proxy for controlling heterospecific effects [57,58]. These variables were chosen to comprehensively represent the factors potentially shaping the abundance distribution of the scavenger guild in the study area. A summary of the specific variables and their sources can be found in Table 1 (see Appendix A for details of data preparation). All units of food availability, human pressure (except urban areas) and heterospecific relationship-related explanatory variables refer to densities, i.e., the quantity or concentration of some abiotic or biotic factor within a given 100 km^2^ grid-cell.

**Table 1:** Explanatory variables used to describe the spatial aggregation patterns and the regional distribution model of the Egyptian vulture in continental Spain. All variables were obtained at a resolution of 10 × 10 km cells (more information in Appendix A).

Acronym	Definition	Source of Information
(1) Habitat		
ALT	Altitude (meters above sea level)	Digital Elevation Model (DEM)
NIC	Cover (%) of non-irrigated crops (e.g., regular annual crops, cereals, leguminous crops)	CORINE Land Cover
IRR	Cover (%) of irrigated crops (e.g., arable, crops, rice fields, non-permanent grass)	CORINE Land Cover
TREE	Cover (%) of permanent crops (e.g., olive groves, orchids, vineyards, fruit trees)	CORINE Land Cover
DEH	Cover (%) of agroforest systems (named *dehesas* in Spain)	CORINE Land Cover
ROC	Cover (%) of bare rocks (e.g., stable rocks with limestone pavements)	CORINE Land Cover
FOR	Cover (%) of forests (e.g., broad-leaved, coniferous, and mixed forests)	CORINE Land Cover
PAS	Cover (%) of pasturelands (e.g., permanent grasslands)	CORINE Land Cover
(2) Food availability		
COW	Number of cows surveyed on national census	National Institute of Statistics (INE)
SHEEP	Number of sheep surveyed on national census	National Institute of Statistics (INE)
LAND	Number of landfills	MAPAMA
SFS	Number of supplementary feeding stations	MAPAMA
(3) Human pressure		
URB	Cover (%) of urban areas (e.g., residential and commercial/industrial buildings, parking lots, small squares)	CORINE Land Cover
WTG	Number of wind turbines	*Asociación Empresarial Eólica* (AEE)
POIS	Number of poison-related mortality events of wild fauna	WWF and SEO/Birdlife [55]
(4) Heterospecific relationship		
GF	Number of breeding pairs of griffon vultures	SEO/Birdlife [59]

## 3. Results

### 3.1. Temporal Variation on Distribution

**Figure 2:**
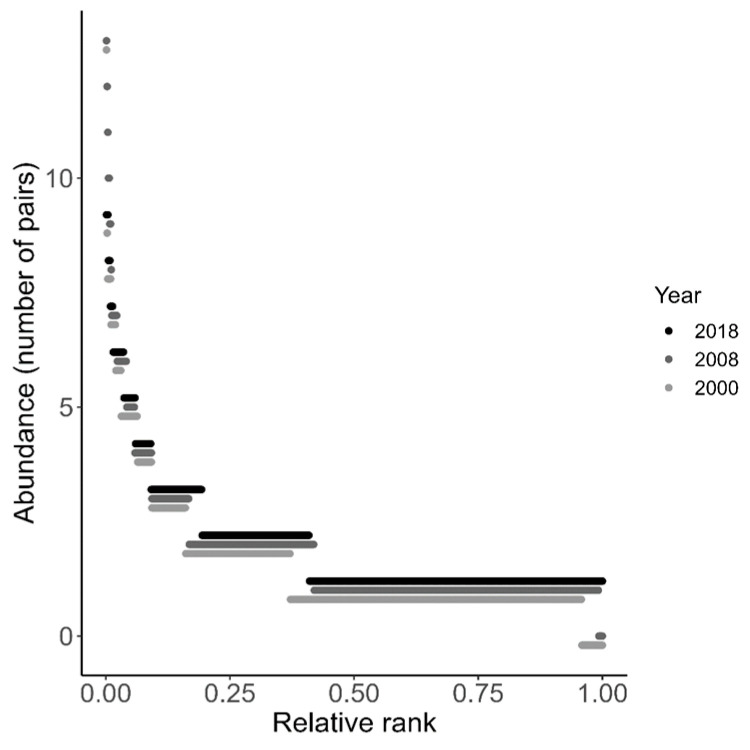
Rank Occupancy–Abundance Profiles (ROAPs) of Spanish national census data of Egyptian vultures in three different years. Local abundance was measured as the number of breeding territories on a 100 km^2^ grid. Relative rank was calculated by dividing the rank descending order of cells by the total number of grid cells in which species has been present at least once during the study period (n = 1033). Grid cells where species were not present in any census.

From year 2000 onwards, censuses (every 8–10 years) showed a slight increase in the total number of breeding pairs of Egyptian vultures in continental Spain, with a total of 1270, 1364, and 1372 pairs in 2000, 2008, and 2018, respectively. Occupancy also increased over the years, with a total of 700, 725, and 731 occupied cells in 2000, 2008, and 2018, respectively. Visual inspection of ROAPs, in combination with the D* statistic, showed an almost exact profile of the three different censuses, indicating that the overall abundance and the frequency of abundances are statistically indistinguishable over the years (Figure 2; Table 2). In addition, the abundance maps for the Egyptian vulture showed a temporal variation in cells despite a similar occupancy and abundance distribution across the study area.

### 3.2. Spatial Variation on Distribution

**Figure 3:**
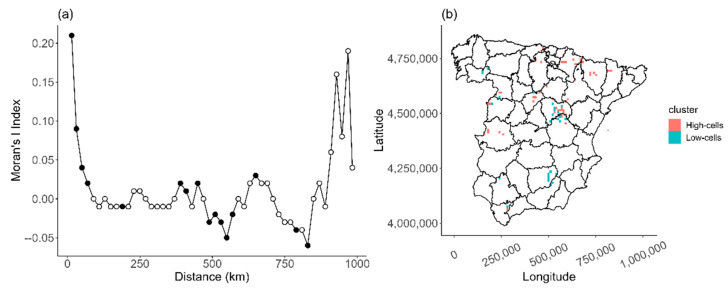
Spatial autocorrelation of the abundance distribution of reproductive pairs of Egyptian vultures in continental Spain in 2018. (**a**) Moran’s I correlogram shows the distance lag between abundances in which spatial autocorrelation is significant (black-filled circles). (**b**) High (red) and Low (blue) cells detected by LISA analysis. High cells represent cells with significantly high abundances compared to neighboring cells, while Low cells represent cells with significantly low abundances compared to neighboring cells.

The autocorrelation analysis showed a strong spatial correlation in the distribution of abundances of the Egyptian vulture. Furthermore, the spatial autocorrelation structure of the abundance distribution remained consistent over the years (see Appendix B). Moran’s test was statistically significant (Moran’s I = 0.075; *P* = 0.001) and the correlogram showed a diminishing positive autocorrelation with increasing distances (Figure 3a). The LISA index of the abundances of breeding pairs in 2018 showed that 57 cells were classified as High-cells, with a mean abundance (±SE) of 5.37 (±0.22) breeding pairs per cell, while 31 cells were classified as Low-cells, with a mean abundance of 0.65 (±0.09) pairs per cell. Meanwhile, the remaining 945 cells were not spatially associated with their neighboring cells in terms of abundance. In addition, the High cells represented 22.3% of the abundance (306 breeding pairs) and occupied 18.12% (5700 km^2^) of the distribution area. The aggregation abundance patterns rarely occurred in isolated cells but were usually a set of two or more cells (Figure 3b).

### 3.3. Local Drivers of Abundance Patterns at Different Spatial Scales

**Figure 4:**
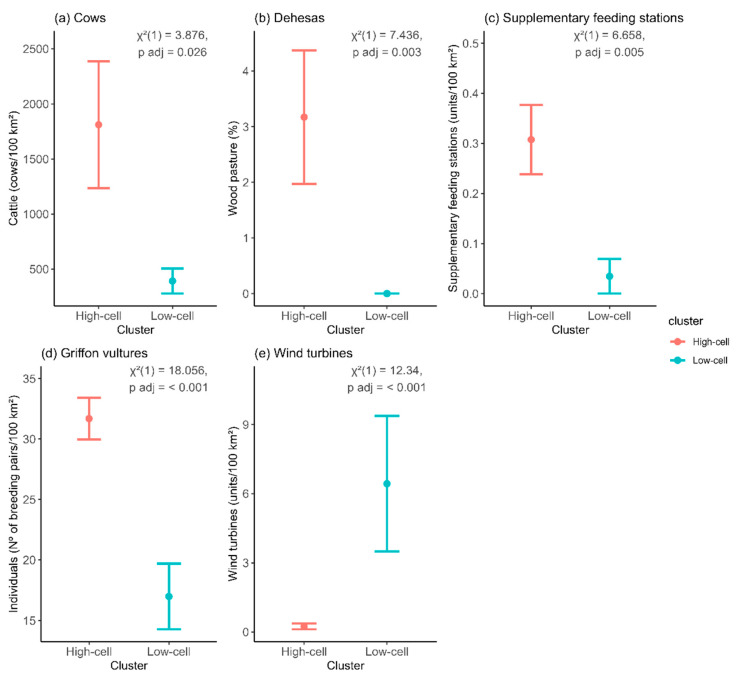
Plots of variables with significant differences between High (H) and Low (L) cells. High cells are areas where environmental features drive a local increase in abundance, while Low cells are areas where environmental features drive a local decrease in abundance. Each variable represents the density of that variable in each 10 × 10 km grid. Plots show mean values of each variable and 95% CIs. We only plotted the variables with significant differences between High and Low cells.

The Kruskal–Wallis comparison between High and Low cells revealed that the densities of cows (ca. heads/100 km^2^), *dehesas* (wood pasture) (%), supplementary feeding stations (units/100 km^2^), and griffon vultures (ca. number of breeding pairs/100 km^2^) were significantly higher in High than in Low cells. Conversely, the number of wind turbines (ca. units/100 km^2^) was significantly lower in the High than in the Low cells, this number being almost seven times higher in the Low cell areas (Figure 4). The remaining variables showed no significant differences (see Appendix C).

By contrast, both GLMs conducted at the regional scale revealed the range of factors affecting the vulture distribution (Table 3). The average models showed statistically positive associations between the abundance of breeding pairs of Egyptian vultures and the abundance of griffon vultures, as well as a weaker association with the number of cows (Appendix D). The model, which included the Egyptian vulture abundance in 2008 as an independent variable, exhibited a better goodness-of-fit (Model 1; pseudo-R^2^ = 0.315) compared to the model that used the abundance in 2000 (Model 2; pseudo-R^2^ = 0.245). Our GLM analysis indicated that the abundance of breeding pairs is primarily explained by previous census variables, while the variation not accounted for by the previous census data is influenced by the abundance of griffon vultures and cows. These latter variables are identified as the principal drivers of changes in Egyptian vulture abundance.

## 4. Discussion

This study presents an analysis of the temporal and spatial variation, as well as the distribution patterns at different scales, of breeding pairs of the endangered Egyptian vulture in one of its global strongholds. Our findings revealed stability in its breeding population in continental Spain after years of continuous decline [31,37]. However, despite this regional stability, significant spatiotemporal variation occurred. The distribution of Egyptian vultures exhibited an aggregated pattern, with the highest abundances concentrated in locations with specific environmental characteristics. This aggregation is a result of scale-dependent factors that shape the population trend. In addition, we identified a hierarchical structure of factors affecting the distribution patterns at two different local and regional scales.

Contrary to our initial expectations, we only found limited significant changes in the regional distribution of the species over time. Both the occupancy and abundance distribution patterns, assessed using ROAPs and D* statistics, exhibited a relatively stable trend during the study period. This stability can be attributed to the intrinsic and consistent fidelity of Egyptian vultures to their breeding territories [60], a characteristic observed in other raptors [61], which ensures that individuals remain in their territories for many years regardless of environmental changes. Moreover, the combination of territorial fidelity and the conspecific attraction of raptor species [62], resulting in the selection of territories near successful conspecific settlements (see *habitat-copying hypothesis* in [63]), probably confers great population stability at a regional scale. However, despite this regional stability, we did detect temporal variability expressed as a large number of cells with low abundance values (e.g., with only one breeding pair) with discontinuous occupancy over time (Figure 1). This observed variability in temporal abundance can be explained by human-related factors (e.g., illegal poisoning; [32,64]) or by demographic stochasticity (i.e., if there are few individuals, the grid cell is more likely to empty).

Additionally, the shape of the ROAP suggested a spatial aggregation of breeding territories. The steep curves indicated that breeding pairs tend to cluster in specific areas, which was confirmed by the LISA analysis that identified cells with a large number of breeding pairs. We observed more cells with low abundances (one breeding pair per cell) than cells with high abundances (five or more breeding pairs per cell), resulting in a heterogeneous distribution pattern. Moreover, this heterogeneity was also supported by our autocorrelation analyses, which revealed clear spatial autocorrelation in the census data over short distances (i.e., 20 km), consistent with patterns observed in other populations (e.g., in Turkey, [65]) and other raptors (e.g., lesser kestrel; [66]). The observed spatial aggregation was found to be a result of scale-dependent factors that shape the abundance distribution. Certain local-level factors such as the presence of wind turbines were associated with lower values of abundance, suggesting they acted as drivers of these patterns. The higher cover of *dehesas* and presence of supplementary feeding stations were associated with more breeding territories, which indicates that these factors favor the study species. However, it is worth noting that these factors only act in specific marginal areas and not throughout continental Spain. For instance, an Egyptian vulture population in southern Spain was affected by wind farm-related mortality during the breeding [34] and migration [67] periods. Additionally, the *dehesas* and agroforestry areas located only in western Spain serve as important foraging habitats for other vulture species due to the higher availability of food compared to other agricultural systems or landscapes [68,69] and support a high relative abundance of livestock grazing and other species (e.g., rabbits) that scavenging birds can exploit. The authors of [70] reported that supplementary feeding stations used as a conservation measure help both the maintenance of the closest breeding territories and breeding success. Nevertheless, these supplementary feeding sites that act as local attractors for high densities of vultures and other scavengers may have detrimental consequences. For instance, supplementary feeding stations can adversely affect the productivity of Pyrenean Bearded vultures (*Gypaetus barbatus*) due to the congregation of non-breeding individuals, leading to a decline in the quality of the reproductive habitat [71].

The main factors associated with changes in abundance at the regional scale over both time and space were griffon vulture and cattle abundances. On the one hand, our results suggest that cattle are one of the main food sources of carrion and feces at local and regional levels for the Egyptian vultures breeding in continental Spain, and play an important role in its distribution [54,72]. In addition, the coprophagous behavior of this species also explains its close association with cows. Egyptian vultures consume cow dung to obtain lutein, a yellow carotenoid responsible for its facial coloration [73] that also plays an important role in its immunological system as an antioxidant [74]. On the other hand, the positive correlation between breeding Egyptian and griffon vultures suggests a heterospecific interaction between these two species that positively impacts the number of Egyptian vulture breeding pairs. Nevertheless, in other studies, the presence of griffon vultures was not associated with the territory occupancy rate of Egyptian vultures as observed in the Balkan Peninsula [23]. In addition, both vulture species probably respond in a similar fashion to specific environmental characteristics, which means that the abundance of griffon vultures will be an indicator of the most suitable habitat for breeding pairs of Egyptian vulture [52,75]. Due to the spatial overlap between these two species, some authors define this interaction as commensalism [76] because (i) both species have similar ecological requirements (e.g., they are both cliff-nesting; [26]) and (ii) given that breeding individuals, regardless of the species, are linked to a breeding area, the abundance of breeding griffon vultures may not only indicate a suitable breeding habitat but also a habitat with food availability [52,76].

Despite the fact that our main aim was to assess the likely causes of changes in the abundance distribution of breeding Egyptian vultures at different spatial scales, other factors relating to human pressure that probably also play an important role in their distribution should not be neglected in future research (e.g., electrocution and/or collision against power lines; [33,34]). Indeed, our results underscore the importance of considering both temporal and spatial variability during the process of generating distribution models. On the one hand, we used temporal population dynamics (i.e., changes between censuses) to capture how the abundance distribution can enhance the subsequent abundance distribution in such a way that the model revealed the suitability of a breeding territory. On the other hand, we took into account spatial autocorrelation in the modelling process because ignoring spatial constraints can lead to inaccurate conclusions (see [9]). To fully understand the changes in endangered species distribution, more research is needed using other approaches, such as Bayesian INLA models, that consider the spatiotemporal variation in species abundance [77].

### Conservation Implications

Our findings reveal the scale-dependent factors that influence the Egyptian vulture breeding population in mainland Spain. At the regional level, these factors require the implementation of global conservation strategies to ensure the species is protected across large areas and to serve as guidelines for developing conservation synergies between neighboring areas. At the local scale, the factors affecting populations or even individuals require specific actions related to the main threats affecting each population. Therefore, it is important to highlight the impact of hierarchical approaches on environmental policies. Thus, successful conservation programs aimed at preserving large vertebrate species over large areas should incorporate efficient local management actions [7,78]. Based on our results, we advocate the development of a national strategy promoting, at the regional level, extensive livestock farming and the abandoning of healthy carcasses (with sanitary control) as an important and unpredictable food source for not only Egyptian vultures but the whole vulture guild [79]. Although this approach is partially implemented through the ZPAEN network (Protection Zones for the Feeding of Necrophagous Species of Community Interest), local administrations use different criteria to establish these zones, which leads to a lack of coordination at the regional scale (see [80]). Additionally, some local actions should be taken to counteract the negative effect of the blades of wind turbines with which certain soaring birds including vultures are prone to collide ([81,82], personal data). Some studies have shown that the strategic placement of wind turbines and appropriate mitigation measures could help minimize the potential negative effects of wind farms on soaring birds while still allowing for the generation of renewable energy [83]. Finally, we believe it is important to underline the importance of grids with a single or few breeding pairs, since the potential for recovery and growth of endangered populations lies in these low-density areas. Conserving small populations allows them to reproduce and expand gradually, and to serve as future sources for repopulating larger areas.

## 5. Conclusions

The breeding Egyptian vulture pairs in continental Spain are generally stable but exhibit spatial variability in their distribution, thereby indicating a hierarchical structure of drivers affecting abundance patterns at different scales. Our data indicate that local-level factors such as the presence of supplementary feeding stations play an important role in the aggregation of breeding pairs. However, the overall stability of the population is mainly driven by the availability of natural food sources, particularly from livestock. Based on the scale-dependent factors influencing the distribution patterns of Egyptian vultures, we recommend the development of a national strategy promoting extensive livestock farming and encouraging the abandoning of healthy carcasses in the field as an important food source for these vultures. In addition, it is important to consider the potential local negative impacts of wind farms and other infrastructures on these species and the need for their strategic placement. Our findings highlight the importance of adopting a holistic approach to conservation efforts that takes into account over time both local- and regional-level factors.

## Figures and Tables

**Table 2:** Egyptian vulture breeding pairs abundance and occupancy changes in Spain in 2000, 2008, and 2018. The D* statistics represent the area under the curve of the ROAPs. *P* is the *p*-value. The abundance change is calculated by subtracting the absolute abundances between years, while the occupancy change is calculated by subtracting the total number of occupied grid cells between years.

Years	D*	*P*	Abundance Change	Occupancy Change
2018–2008	0.992	0.648	8	6
2008–2000	0.970	0.615	94	25
2018–2000	0.918	0.640	102	31

**Table 3:** Top 10 competing models for GLM from abundance distribution of Egyptian vultures in continental Spain. The abundance of 2008 (NP08) was included as an independent variable in Model 1 and the abundance of 2000 (NP00) was included as an independent variable in Model 2. Y- and X-related variables correspond to the third-polynomial spatial terms of the model.

Model	Variables	df	Loglik	AICc	Delta	Weight
1.1.	COW + GF + NP08 + Y + Y^2^	7	−1365.67	2745.44	0.000	0.0046
1.2.	COW + GF + NP08 + Y + Y^3^	7	−1365.69	2745.50	0.058	0.0045
1.3.	COW + GF + NP08 + Y^2^ + Y^3^	7	−1365.73	2745.57	0.127	0.0043
1.4.	COW + GF + SFS + NP08 + Y + Y^2^	8	−1365.04	2746.22	0.785	0.0031
1.5.	COW + GF + SFS + NP08 + Y + Y^3^	8	−1365.07	2746.27	0.834	0.0030
1.6.	COW + GF + SFS + NP08 + Y^2^ + Y^3^	8	−1365.10	2746.33	0.895	0.0029
1.7.	COW + GF + NP08 + X + X^2^Y + XY + XY^2^	9	−1364.14	2746.46	1.023	0.0028
1.8.	COW + DEH + GF + NP08 + Y + Y^2^	8	−1365.17	2746.48	1.039	0.0027
1.9.	COW + GF + NP08 + WTG + Y + Y^2^	8	−1365.19	2746.52	1.080	0.0027
1.10.	COW + GF + NP08 + X + X^2^ + XY + XY^2^	9	−1364.18	2746.54	1.099	0.0027
2.1.	COW + GF + NP00 + Y + Y^2^	7	−1415.04	2844.19	0.000	0.004
2.2.	COW + GF + NP00 + Y + Y^3^	7	−1415.08	2844.27	0.076	0.004
2.3.	COW + GF + NP00 + Y^2^ + Y^3^	7	−1415.13	2844.36	0.168	0.004
2.4.	COW + GF + NP00 + WTG + Y + Y^2^	8	−1414.27	2844.67	0.479	0.003
2.5.	COW + GF + NP00 + WTG + Y + Y^3^	8	−1414.29	2844.72	0.527	0.003
2.6.	COW + GF + NP00 + WTG + Y^2^ + Y^3^	8	−1414.32	2844.78	0.589	0.003
2.7.	COW + DEH + GF + NP00 + Y + Y^2^	8	−1414.41	2844.97	0.773	0.003
2.8.	COW + DEH + GF + NP00 + Y + Y^3^	8	−1414.48	2845.10	0.903	0.003
2.9.	COW + DEH + GF + NP00 + Y^2^ + Y^3^	8	−1414.55	2845.25	1.052	0.002
2.10.	COW + DEH + GF + NP00 + WTG + Y + Y^2^	9	−1413.57	2845.33	1.132	0.002

**Figure A1:**
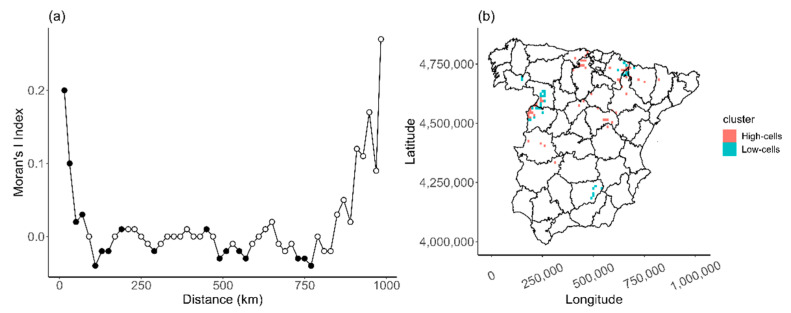
Spatial autocorrelation of the abundance distribution of reproductive pairs of Egyptian vultures in continental Spain in 2008. (**a**) Moran’s I correlogram shows the distance lag between abundances in which spatial autocorrelation is significant (black-filled circles). (**b**) High (red) and Low (blue) cells detected by LISA analysis. High cells represent cells with significantly high abundances compared to neighboring cells, while Low cells represent cells with significantly low abundances compared to neighboring cells.

**Figure A2:**
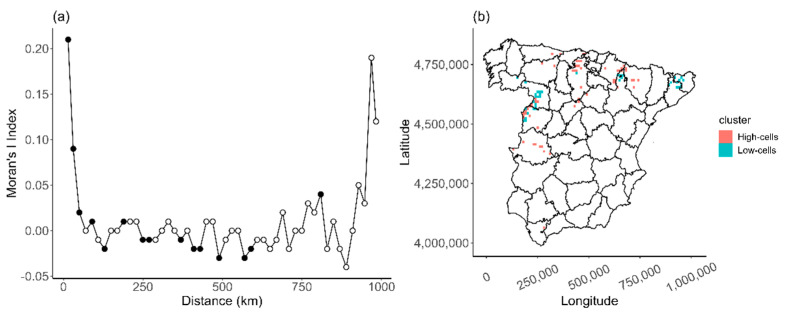
Spatial autocorrelation of the abundance distribution of reproductive pairs of Egyptian vultures in continental Spain in 2000. (**a**) Moran’s I correlogram shows the distance lag between abundances in which spatial autocorrelation is significant (black-filled circles). (**b**) High (red) and Low (blue) cells detected by LISA analysis. High cells represent cells with significantly high abundances compared to neighboring cells, while Low cells represent cells with significantly low abundances compared to neighboring cells.

## Data Availability

No new data were created or analyzed in this study. Data sharing is not applicable to this article.

## Appendix A

### Appendix A.1. Digital Elevation Model

The elevation data were obtained from the 25 m resolution Digital Elevation Model provided by the National Institute of Geography (www.ign.es, accessed on 19 December 2019). To align the resolution with our 100 km^2^ abundance data, we resampled the raster by applying a grid with fewer pixels. To do so, we used the *resample*() function of the raster R package and a bilinear interpolation method that uses the distance-weighted average of the four nearest pixel values to estimate a fresh pixel value.

### Appendix A.2. CORINE Land Cover Maps

We used the 2018 CORINE land cover maps from Copernicus (www.land.copernicus.eu, accessed on 16 October 2019) to obtain seven variables relating to habitat and one variable relating to human pressure. The CORINE maps include 44 land-cover classes, which we reclassified into 8 different cover classes: non-irrigated crops, irrigated crops, tree crops, agroforest systems known as *dehesas*, bare rock, forests, pasturelands, and urban areas. Table A1 shows the reassigned land-cover classes. Next, we calculated the percentage of the land cover by converting each reclassified land-cover class into polygon shapefiles, extracting the portion of the land cover in each cell, and finally rasterizing the layer with the proportion of land cover of each cell using the elevation layer as the raster base.

**Table A1:** Reclassification of CORINE land covers (CLC) with the new categories URB: urban areas; NIC: non-irrigated crops; IRR: irrigated crops; TREE: permanent crops; PAS: pasturelands; DEH: agroforestal systems (*dehesas*), and FOR: forests. Some categories of CORINE Land Cover were not reassigned to a new category. Modified table CORINE Land Use Covers 2018 legend (land.copernicus.eu, access on 16 October 2019).

CLC Level 1	CLC Level 2	CLC Level 3	New Code
Artificial surfaces	Urban fabric	Continuous urban fabric	URB
		Discontinuous urban fabric	URB
	Industrial, commercial, and transport units	Industrial or commercial units	URB
		Road and rail networks and associated land	URB
		Port area	URB
		Airports	URB
	Mine, dump, and construction sites	Mineral extraction sites	URB
		Dump sites	URB
		Construction sites	URB
	Artificial, non-agricultural vegetated areas	Green urban areas	URB
		Sport and leisure facilities	URB
Agricultural areas	Arable land	Non-irrigated arable land	NIC
		Permanently irrigated land	IRR
		Rice fields	IRR
	Permanent crops	Vineyards	TREE
		Fruit trees and berry plantations	TREE
		Olive groves	TREE
	Pastures	Pastures	PAS
	Heterogeneous agricultural areas	Annual crops associated with permanent crops	NIC
		Complex cultivation patterns	NIC
		Land principally occupied by agriculture, with significant areas of natural vegetation	NIC
		Agroforestal areas	DEH
Forest and semi natural areas	Forest	Broad-leaf forest	FOR
		Coniferous forest	FOR
		Mixed forest	FOR
	Scrub and/or herbaceous vegetation associations	Natural grasslands	PAS
		Moors and heathland	-
		Sclerophyllous vegetation	-
		Transitional woodland–shrub	-
	Open spaces with little or no vegetation	Beaches, dunes, sands	ROC
		Bare rocks	ROC
		Sparsely vegetated areas	ROC
		Burnt areas	-
		Glaciers and perpetual snow	-
Wetlands	Inland wetlands	Inland marshes	-
		Peat bogs	-
	Maritime wetlands	Salt marshes	-
		Salines	-
		Intertidal flats	-
Water bodies	Inland waters	Water courses	-
		Water bodies	-
	Marine waters	Coastal lagoons	-
		Estuaries	-
		Sea and ocean	-

### Appendix A.3. INE

Food availability information (i.e., cows and sheep) was obtained from the agricultural census of Spain (www.ine.es, accessed on 16 December 2019), which calculates the number of different domestic animals in each municipality. We translated the density of domestic animals from each municipality into spatial information. Then, we rasterized the spatial information using a bilinear interpolation (see above) using the elevation raster as a base layer.

### Appendix A.4. MAPAMA

Data on landfills and supplementary feeding stations, also used as food resources by Egyptian vultures, were obtained from the Ministry of Agriculture, Fisheries, Food and Environment (MAPAMA; www.mapa.god.es, accessed on 10 October 2019). Landfill locations were used to create a raster of landfill density. As data on landfills in Catalonia and Valencia were not available from MAPAMA, we obtained this information from the Catalan Waste Agency (www.residus.gencat.cat, accessed on 22 October 2019) and the Environment Department of the Generalitat Valenciana (www.agroambient.gva, accessed on 22 October 2019), respectively. We also obtained the geographic locations of all supplementary feeding stations and verified their operational status in 2000–2018. Information on active supplementary feeding stations was included in the data using the same procedure as for landfills.

### Appendix A.5. Asociación Empresarial Eólica (AEE)

We obtained the number of wind turbines per 10 × 10 km cell from the locations of national wind farms and their corresponding number of wind turbines (available at www.aeeolica.org, accessed on 14 October 2019). We first obtained the geographic locations of all wind turbines and then created a raster by rasterizing the information on the density of wind turbines in each cell using the elevation layer as a base map.

### Appendix A.6. The Poison-Related Mortality Event Database from SEO/Birdlife and WWF

The number of poison-related mortality events was calculated using the “El veneno en España “[57] database. We considered a poison-related mortality event to be the use of any chemical substance that causes the death of wildlife after ingestion. We assumed that several dead wild animals found at the same location within a 15-day period represented the same poison-related mortality event. We obtained the location of each poison-related mortality event and calculated the number of poison-related mortality events per 10 × 10 km cell.

### Appendix A.7. Griffon Vulture National Census

We used the same procedure as for the Egyptian vulture to obtain the number of breeding pairs of griffon vultures (*Gyps fulvus*). For each communal breeding area or colony, we recorded the location and status (occupied vs. unoccupied). To obtain the abundance distribution, we incorporated these locations into a 10 × 10 km grid-cell and calculated the abundance in each cell by summing the locations of the confirmed communal breeding areas.

## Appendix C

**Table A2:** Kruskal–Wallis test of no significant differences between High cells and Low cells. *P* is the *p*-value. *P adj* is the *p*-value adjusted using the Bonferroni correction.

Variables	H	df	*P*	*P adj*
(1) Habitat				
ALT	0.069	1	0.792	0.3958
NIC	16.748	1	0.650	0.325
IRR	2.654	1	0.103	0.052
TREE	0.842	1	0.359	0.179
ROC	62.978	1	0.903	0.451
FOR	93.876	1	0.761	0.381
PAS	0.400	1	0.527	0.264
(2) Food availability				
SHEEP	1.444	1	0.230	0.115
LAND	3.648	1	0.581	0.291
(3) Human pressure				
URB	2.018	1	0.155	0.078
POIS	2.449	1	0.118	0.059

## Appendix D

**Table A3:** Estimates for average GLM (selection based on those with the lowest AICc scores, with a ΔAIC threshold of <2) describing abundance distribution of Egyptian vultures in continental Spain. Two models are specified: Model 1 incorporates the 2008 Egyptian vulture abundances (NP08) as a predictor variable and Model 2 incorporates the 2000 abundances (NP00). *P* is the *p*-value. Significant *p*-values < 0.05 are **in bold**.

	Variables	E	SE	Adj SE	Z	*P*
Model-1	Intercept	−36.790	74.020	74.080	0.497	0.619
	NP08	0.196	0.012	0.012	16.185	**<0.001**
	COW	1.647 × 10^−5^	6.569 × 10^−6^	1.647 × 10^−5^	2.504	**0.012**
	GF	0.141	0.020	0.020	6.931	**<0.001**
	MUL	−0.042	0.037	0.037	1.116	0.264
	DEH	−0.003	0.003	0.003	0.981	0.327
	WTG	−0.002	0.002	0.002	1.059	0.290
	y	2.86 × 10^−5^	6.46 × 10^−5^	6.47 × 10^−5^	0.442	0.658
	y^2^	−1.91 × 10^−12^	1.48 × 10^−11^	1.48 × 10^−11^	0.129	0.898
	y^3^	−1.96 × 10^−19^	1.19 × 10^−18^	1.19 × 10^−18^	0.164	0.869
	x	−2.19 × 10^−4^	5.90 × 10^−5^	5.91 × 10^−5^	3.707	**<0.001**
	x^2^y	−2.76 × 10^−19^	1.85 × 10^−19^	1.86 × 10^−19^	1.488	0.137
	xy	9.56 × 10^−11^	2.55 × 10^−11^	2.56 × 10^−11^	3.741	**<0.001**
	xy^2^	−1.04 × 10^−17^	2.76 × 10^−18^	2.76 × 10^−18^	3.762	**<0.001**
	x^2^	−1.30 × 10^−12^	8.80 × 10^−13^	8.81 × 10^−13^	1.472	0.141
	x^3^	−4.23 × 10^−19^	5.32 × 10^−19^	5.33 × 10^−19^	0.795	0.427
Model-2	Intercept	−68.530	56.810	56.850	1.205	0.228
	NP00	0.166	0.015	0.015	10.936	**<0.001**
	COW	0.000	0.000	0.000	2.492	**0.013**
	GF	0.182	0.020	0.020	9.097	**<0.001**
	MUL	−0.026	0.035	0.035	0.726	0.468
	DEH	−0.004	0.003	0.003	1.348	0.178
	WTG	−0.003	0.002	0.002	1.214	0.225
	y	3.46 × 10^−5^	4.03 × 10^−5^	4.03 × 10^−5^	0.859	0.390
	y^2^	−7.19 × 10^−13^	9.99 × 10^−12^	1.00 × 10^−11^	0.072	0.943
	y^3^	−4.71 × 10^−19^	7.20 × 10^−19^	7.21 × 10^−19^	0.653	0.514
	x	−4.36 × 10^−5^	1.06 × 10^−4^	1.06 × 10^−4^	0.412	0.681
	x^2^y	1.89 × 10^−11^	4.61 × 10^−11^	4.61 × 10^−11^	0.410	0.682
	xy	−2.07 × 10^−18^	5.00 × 10^−18^	5.00 × 10^−18^	0.414	0.679
	xy^2^	−1.50 × 10^−13^	1.67 × 10^−13^	1.67 × 10^−13^	0.896	0.371
	x^2^	−3.10 × 10^−20^	3.58 × 10^−20^	3.58 × 10^−20^	0.867	0.386
	x^3^	−1.34 × 10^−19^	1.85 × 10^−19^	1.85 × 10^−19^	0.726	0.468
